# Vertebrate herbivory weakens directional selection for earlier emergence in competition

**DOI:** 10.1002/evl3.222

**Published:** 2021-03-25

**Authors:** Joseph Waterton, Elsa E. Cleland

**Affiliations:** ^1^ Department of Ecology, Behavior, and Evolution Section University of California San Diego La Jolla California 92093; ^2^ Current Address: Department of Biology Indiana University Bloomington Indiana 47405

**Keywords:** Competition, emergence timing, herbivory, phenology, phenotypic selection

## Abstract

The timing of seedling emergence is strongly linked with fitness because it determines the biotic and abiotic environment experienced by plants in this vulnerable life stage. Experiments and observations consistently find that earlier‐emerging plants have a competitive advantage over those emerging later. However, substantial genetic and phenotypic variation in emergence timing is harbored within and among plant populations, making it important to characterize the selective agents—including biotic interactions—that contribute to this variation. In seasonal herbaceous communities, we hypothesized that consumption of early‐emerging individuals by vertebrates could weaken the strength of directional selection for earlier emergence in competitive environments. To investigate this, we carried out phenotypic selection analyses on emergence timing in two California grass species, the native *Stipa pulchra* and non‐native *Bromus diandrus*, growing in intraspecific competitive neighborhoods with and without vertebrate herbivore exclusion. Vertebrate herbivores consistently weakened directional selection for earlier emergence. Our results demonstrate that vertebrate herbivores play an underappreciated selective role on phenology in plant populations, with implications for contemporary evolution, such as the potential of species to adapt to global environmental changes.

**Impact Summary**

The timing of phenological events, such as emergence and flowering, is a critical component of adaptation to seasonal environments. For plants, the timing of seedling emergence is the first phenological trait expressed within a growing season, yet the role of trophic interactions in shaping natural selection on emergence timing is poorly understood. We show that generalist vertebrate herbivory weakens directional selection favoring earlier emergence for plants growing in competition. Our results demonstrate that herbivory plays an underappreciated role in shaping natural selection on emergence timing in seasonal environments, with the potential to mediate evolutionary responses to global environmental changes.

Phenological traits, such as the timing of emergence, diapause, and migration, are critical to organismal adaptation in seasonal environments. In plants, phenology strongly influences individual fitness (Kalisz 1986; Donohue et al. 2005; Bogdziewicz et al. 2020; Chapurlat et al. 2020), and these individual‐level responses can scale up to alter population dynamics (Keller and Shea 2020) and species’ range limits (Benning et al. 2019). Furthermore, plant phenology structures interactions with other species in biological communities, including herbivores (Post and Forchhammer 2008), pollinators (Kudo and Ida 2013), and heterospecific competitors (Alexander and Levine 2019). Climate plays a key role in shaping plant phenology, through both adaptive evolution to long‐term climatic conditions and plasticity in response to interannual variation in them (Anderson et al. 2012; Munson and Sher 2015; Ågren et al. 2017). Biotic interactions also mediate selection on plant phenology; for example, selection on flowering phenology is influenced by pollinators (Sletvold et al. 2010; Chapurlat et al. 2015) and predispersal seed predators (Pilson 2000; Valdés and Ehrlén 2017). Such biotic‐mediated selection on phenology could reinforce or oppose direct selection imposed by local climate (Van der Putten et al. 2010), and thus characterizing it is crucial for interpreting patterns of genotypic and phenotypic variation in plant phenology and predicting shifts in response to climate change.

The timing of seedling emergence is the earliest phenological trait expressed in a growing season; this determines the biotic and abiotic environment experienced by plants, influencing both fitness and patterns of selection on traits expressed subsequently (Donohue 2002). Earlier emergence within growing seasons is consistently associated with higher fitness (Verdú and Traveset 2005) because it allows longer periods of resource uptake as well as the ability to pre‐empt resources and competitively suppress later‐arriving individuals (i.e., priority effects) (Vannette and Fukami 2014). Despite this consistent growth advantage of earlier emergence, emergence timing is notable for displaying substantial genetic and phenotypic variation within populations (Simons and Johnston 2006) and among populations (reviewed in Cochrane et al. 2015), as well as substantial phenotypic variation within genotypes (Galloway 2001; Simons and Johnston 2006). Like other phenological traits, climate is well established as an important selective agent on emergence timing. For example, early‐season abiotic stress, such as drought and frost, can favor later emergence within a growing season (Shimono and Kudo 2003; Wainwright et al. 2012), and studies have documented later emergence in populations that more frequently experience such conditions (Meyer and Monsen 1991; Torres‐Martínez et al. 2017). Furthermore, interannual variation in climate has been linked to variable selection on emergence time (Kalisz 1986), which is regarded as a key driver of the evolution of diversified emergence timing within genotypes (Simons and Johnston 2006; Simons 2009). Although research has largely focused on the role of local climate as a selective agent on emergence timing, much less is known about how biotic interactions, such as herbivory, influence the evolution of this key phenological trait.

In seasonal herbaceous communities, grazing by vertebrate herbivores might, through several potential mechanisms, weaken directional selection for earlier emergence in competitive environments. First, plants that emerge earlier might experience early‐season herbivory that later‐emerging individuals can escape by remaining as seeds in the soil. Second, the size advantage gained from earlier emergence could lead to selective grazing by increasing apparency or accessibility to herbivores throughout the growing season (Louthan et al. 2014; Thomann et al. 2018). Third, even without selective grazing on larger plants, grazing could lead to the competitive release of smaller, later‐emerging plants by reducing light limitation (Borer et al. 2014). Across species, earlier emergence can increase susceptibility to vertebrate herbivores, particularly during the early period of the growing season when impacts can be greatest (Waterton and Cleland 2016). However, whether a similar process weakens directional selection for earlier emergence within species is unknown. We tested the hypothesis that vertebrate herbivores weaken directional selection for earlier emergence by carrying out a field experiment in which we characterized phenotypic selection on emergence time in two California grasses, the native perennial *Stipa pulchra* (Hitchc.) Barkworth and the non‐native annual *Bromus diandrus* (Roth), growing under intraspecific competition with and without the exclusion of vertebrate herbivores.

## Methods

### STUDY SITE

We conducted the experiment on an experimental field at the University of California San Diego Biological Field Station (32.885° N, 117.230° W) during a single growing season between February and June 2018, which was timed to occur contemporaneously with natural emergence and growth at the study site. The site is surrounded by coastal sage scrub habitat and has a Mediterranean‐type climate: most annual precipitation typically falls between November and May, the onset of which initiates the emergence and subsequent growth of seedlings, including both focal species (Bartolome and Gemmill 1981; Young et al. 1981). However, unusually dry winter conditions delayed the emergence of naturally occurring seedlings at the site until after a large rain event on 9 and 10 January 2018. Data from the PRISM Climate Group database (prism.oregonstate.edu/) showed that January‐June precipitation at the site was 41% lower than the 30‐year average (Fig. S1). The site is flat, regularly tilled, and is classified as having sandy clay loam soil. The dominant plant species are non‐native annuals, including *Hordeum murinum*, *Erodium cicutarium*, and *Malva parviflora*. Both focal species, *S. pulchra* and *B. diandrus*, are uncommon at the experimental site, making it feasible to identify planted individuals. Generalist mammalian herbivores observed at the site include the brush rabbit (*Sylvilagus bachmani*), the desert cottontail (*Sylvilagus audubonii*), California ground squirrels (*Otospermophilus beecheyi*), and several neotomine mouse species. Also present are numerous bird species that are important consumers of seedlings in coastal sage scrub (Litle et al. 2019). No data were available on the density of herbivores during the experiment or at other times.

### STUDY SPECIES

*Stipa pulchra* (purple needlegrass) is a perennial bunchgrass native to California that is found in woodland, chaparral, and grassland from Baja California to northern California (Baldwin et al. 2012). *Stipa pulchra* ceases vegetative growth during summer drought and recommences with the arrival of winter rains (Laude 1953). *Stipa pulchra* can survive for up to 100 years (Hamilton et al. 2002), but native vertebrate herbivores reduce *S. pulchra* survival and fitness in both seedlings and adults (Orrock et al. 2009). A study of neutral genetic markers shows that *S. pulchra* is highly self‐fertilizing (reported selfing rate ≈ 1) (Larson et al. 2001). *Stipa pulchra* typically do not reproduce in the first season of growth, therefore we did not measure reproductive output. We analyzed fitness in *S. pulchra* using end‐of‐growing‐season aboveground biomass (hereafter “biomass,” see below). Aboveground biomass in *S. pulchra* is positively correlated with the probability of surviving through summer drought (Allen 1995). Furthermore, in another experiment at the same site that lasted two growing seasons, *S. pulchra* aboveground biomass was also strongly positively correlated with total seed production (*r* = 0.87, *P* < 0.001) (J. Waterton, unpubl. ms.); therefore, we regarded it to be an appropriate and relevant fitness component while emphasizing that it does not represent lifetime fitness.

*Bromus diandrus* is an annual grass native to Eurasia that has become naturalized in California since European settlement in the 18th century (Jackson 1985). This species is particularly dominant in disturbed areas such as abandoned agricultural fields (Stromberg and Griffin 1996). *Bromus diandrus* is also highly self‐fertilizing (reported selfing rate > 0.99) (Kon and Blacklow 1990). Due to its annuality, we analyzed fitness in *B. diandrus* using the total weight of seeds produced per individual (hereafter “fecundity,” see below), a measure of total female lifetime fitness.

### HERBIVORE EXCLUSION EXPERIMENT

We used field‐collected seeds from naturally occurring populations, with *B. diandrus* sourced from Elliott Chaparral Reserve (32.889° N, 117.091° W) and *S. pulchra* sourced from Sedgwick Reserve (34.692° N 120.043° W). We collected seeds following the protocol of Knapp and Rice (1998), with maternal plants (hereafter referred to as maternal lines) spaced at least 5 m apart to avoid collecting from clonal neighbors (Dyer and Rice 1997). We collected seeds in April 2015 and stored them at 4°C until use. For each of 16 maternal lines in each focal species, we selected 32 seeds that outwardly appeared viable and randomly assigned half to each of two caging treatments: “herbivory” or “exclusion.” This resulted in 256 seeds for each focal species in each caging treatment (1024 seeds in total). We weighed each seed to the nearest 0.01 mg for inclusion in phenotypic selection analyses because seed mass can influence emergence time (Simons and Johnston 2000; Susko and Lovett‐Doust 2000) and influence fitness directly (Simons and Johnston 2000; Halpern 2005).

**Figure 1:**
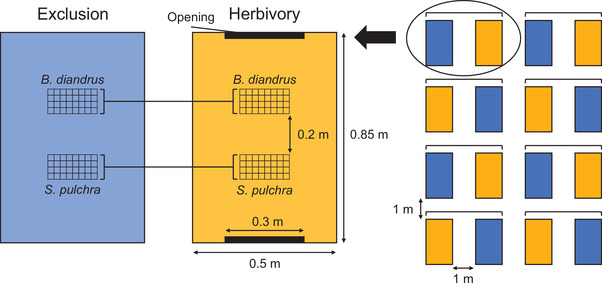
Layout of experimental plots. The right‐hand side shows the layout of plots with brackets connecting plots in a block. The left‐hand side shows a single block, with brackets connecting subplots of 32 seeds that share planting arrangements of maternal lines.

In January 2018, we established sixteen 85 cm × 50 cm plots, eight for each caging treatment, arranged in rows of four, with 1 m spacing between adjacent plots. We watered and tilled plots to germinate and remove the standing seedbank. We placed covered cages made from 6.3 mm hardware cloth around plots and cut 30 cm × 30 cm openings in the two shorter sides of cages in the herbivory treatment. Cages in both treatments intercepted approximately 27% of photosynthetically active radiation (PAR), as measured on a cloudless day at noon with an AccuPAR LP‐80 PAR Ceptometer (Decagon Devices Inc., Washington, USA). We arranged plots so that caging treatments alternated along each row to minimize potential spatial effects on plant growth and rates of herbivory. Within plots, we installed two separate 4 × 8 planting grids made from 1.25 cm hardware cloth (one for each focal species), placed 20 cm apart in the center of each plot (hereafter “subplot”). Figure 1 shows the layout of experimental plots.

We started the experiment on 1 February 2018, approximately 2 weeks after the first seedlings were observed emerging naturally at the field site. In each plot, we planted 32 seeds of each focal species (two seeds per maternal line) into each of the two subplots, such that each subplot contained a single species with one seed in each grid square (a planting density of 6400 seeds per square meter) (Fig. 1). This density is within the natural range that has been observed for California grasses (including *B. diandrus*) and thus represents realistic levels of intraspecific competition (Young et al. 1981). For each species, we generated eight random planting arrangements of maternal lines within subplots that we assigned to sets of two adjacent herbivory and exclusion treatment plots (hereafter “block”) (Fig. 1). We watered plots immediately after planting to initiate germination and then watered plots daily until the first observed rain event on February 21. We monitored plots daily, recording for each individual the date of emergence and the date of first observed vertebrate herbivore damage (i.e., grazing). We did not quantify the extent of damage for each individual but recorded only the presence/absence of damage that we could confidently identify as herbivory (e.g., clipped tissue, bite marks, contemporaneous appearance of scat). We recorded only the date of first damage as we were unable to reliably identify new instances of damage on plants that had previously been eaten. We monitored damage until 25 April, after which the high density of plants in subplots precluded the identification of new damage; thus, we refer to “early‐season damage” only in our analyses and discussion. We observed no evidence of any vertebrate herbivore damage or disturbance in the exclusion treatment. As expected, no *S. pulchra* individuals flowered. We harvested aboveground biomass of *S. pulchra* on 7 June. We harvested *B. diandrus* seeds on 13 June by which point plants had senesced and seeds were mature but still firmly attached to culms. We dried samples for 3 days at 40°C before weighing to the nearest 1 mg. Additional details of experimental methods are provided in the Supporting Information.

### STATISTICAL ANALYSES

We conducted all statistical analyses separately for each focal species, using R version 3.6.1 (R Core Team 2019). We used a generalized linear mixed model (GLMM) with a binomial distribution and a logit link to test whether the probability of emerging was predicted by caging treatment, seed mass, and their interaction, with block and maternal line as random effects. We used a linear mixed model (LMM) to test how emergence time (log‐transformed to improve normality of residuals) was influenced by caging treatment, seed mass, and their interaction, with block and maternal line as random effects. We used a binomial GLMM with a logit link to test how the probability of experiencing early‐season herbivore damage in the herbivory treatment was influenced by emergence time and seed mass, with block and maternal line as random effects.

We used hurdle GLMMs to separately analyze two components of fitness in each focal species (Wadgymar et al. 2015). For the perennial *S. pulchra*, we analyzed (1) survival to biomass harvest (“survival”) and (2) aboveground biomass of individuals that survived (“biomass”). For the annual *B. diandrus*, we analyzed (1) survival to seed production (“survival”) and (2) total mass of seeds produced (“fecundity”). We analyzed survival components using binomial GLMMs with a logit link (zero part). Nonzero values of fecundity and biomass were overdispersed and therefore we analyzed these with zero‐truncated negative binomial GLMMs with a log link (nonzero part).

To test whether mean values of each fitness component differed between caging treatments, we fit zero and nonzero hurdle GLMM parts with relevant fitness components predicted by caging treatment, with block and maternal line as random effects. To evaluate direct linear (*β*_i_) and quadratic (*γ*_ii_) phenotypic selection on traits, and how these differed between caging treatments, we fit zero and nonzero GLMM model parts with relevant fitness components predicted by traits (emergence time and seed mass), caging treatment, and trait × caging treatment interactions, with block and maternal line as random effects. For selection analyses, we standardized trait values across caging treatments to a mean of zero and standard deviation of one. We evaluated linear selection with models containing linear trait effects only, and quadratic selection with models that contained linear and quadratic trait effects. We did not consider the interaction between seed mass and emergence time (i.e., the cross‐product, *γ*_ij_) because no models contained more than one significant quadratic trait term (see Results). We report—and use as the basis for figures and discussion—the coefficients from hurdle GLMMs as the most statistically sound estimates of direct linear and quadratic selection acting on traits in each treatment (Wadgymar et al. 2015). We note that these are not equivalent to standardized selection gradients from the previously standard multiple regression approach (Lande and Arnold 1983). Significant trait × caging treatment interactions indicate that selection differs between caging treatments (i.e., significant herbivore‐mediated selection), in which case we then tested the significance of selection in each caging treatment using separate models. To accompany tests of trait × caging treatment interactions, we quantified herbivore‐mediated selection (Δ*β*_i(Herb)_ or Δ*γ*_ii(Herb)_), by subtracting selection gradients in the exclusion treatment (*β*_i(Exc)_ or *γ*_ii(Exc)_) from selection gradients in the herbivory treatment (*β*_i(Herb)_ or *γ*_ii(Herb)_), such that Δ*β*_i(Herb)_ = *β*_i(Herb)_ – *β*_i(Exc)_ and Δ*γ*_ii(Herb)_ = *γ*_ii(Herb)_ – *γ*_ii(Exc)_ (Sletvold et al. 2010; Chapurlat et al. 2015).

We calculated Spearman's rank correlations of emergence time and seed mass in each caging treatment to confirm that models containing both traits as predictors were unaffected by severe multicollinearity (*r*_s_ ≤ −0.70 or ≥ 0.70) (Mitchell‐Olds and Shaw 1987; cf. Warwell and Shaw 2019); *r*_s_ in both *S. pulchra* (exclusion *r*_s_ = −0.27, herbivory *r*_s_ = −0.26) and *B. diandrus* (exclusion *r*_s_ = −0.34, herbivory *r*_s_ = −0.39) indicated severe multicollinearity was not present. To confirm the appropriateness of the zero‐truncated negative binomial distribution for nonzero hurdle GLMM parts, we used the *simulateResiduals* function in the package *DHARMa* (Hartig and Lohse 2019). We fit LMMs using the *lmer* function in the package *lme4* (Bates et al. 2015) and GLMMs using the *glmmTMB* function in the package *glmmTMB* (Brooks et al. 2017). We evaluated the significance of fixed effects in LMMs and GLMMs with Type II Wald chi‐square tests using the *Anova* function in the package *car* (Fox and Weisberg 2011).

## Results

### EMERGENCE AND SEED MASS OF FOCAL INDIVIDUALS

Of the 512 seeds planted per species, 495 *S. pulchra* individuals (96.7%) and 475 *B. diandrus* individuals (92.8%) emerged in total. Heavier seeds were more likely to emerge in both *S. pulchra* (*χ* ^2^_(1)_ = 14.4, *P* < 0.001; Fig. S2A) and *B. diandrus* (*χ* ^2^_(1)_ = 13.1, *P* < 0.001; Fig. S2B). Caging treatment did not affect emergence probability in *S. pulchra* (*χ* ^2^_(1)_ = 2.29, *P* = 0.13; Fig. S2A) or *B. diandrus* (*χ* ^2^_(1)_ = 0.030, *P* = 0.86; Fig. S2B), and there was no interaction between seed mass and caging treatment in *S. pulchra* (*χ* ^2^_(1)_ = 0.066, *P* = 0.80; Fig. S2A) or *B. diandrus* (*χ* ^2^_(1)_ = 0.004, *P* = 0.95; Fig. S2B).

**Figure 2:**
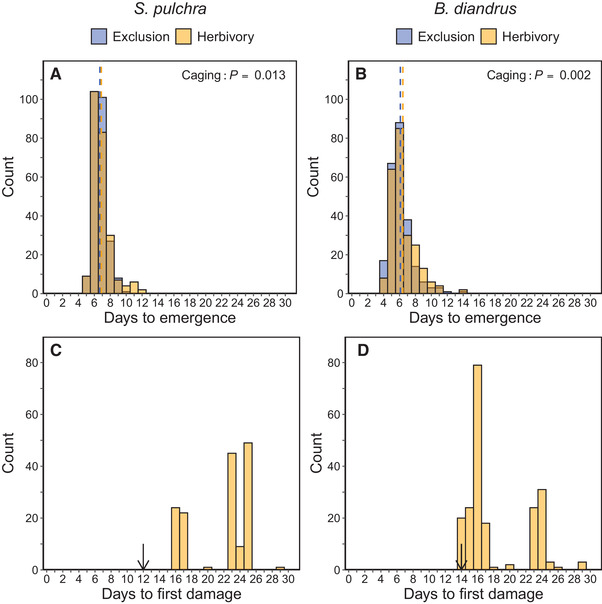
Histograms of emergence time and days to first observed damage. (A and B) Histograms of days to emergence in caging treatments in (A) *S. pulchra* (*n* = 495) and (B) *B. diandrus* (*n* = 475). Dashed lines represent mean values in each caging treatment. *P*‐values are from linear mixed models of emergence time (log‐transformed) predicted by caging treatment, seed mass, and their interaction, with block and maternal line specified as random effects. (C and D) Histograms of days to first observed damage in the herbivory treatment in (C) *S. pulchra* (*n* = 245) and (D) *B. diandrus* (*n* = 237). We calculated days to first damage from the start of the experiment on 1 February. Arrows represent the final day in which emergence was observed for each species. We monitored herbivore damage until day 83 (25 April), after which the high density of plants in subplots precluded the reliable identification of new damage instances.

*Stipa pulchra* emerged over the span of 8 days, beginning 5 days after planting (Fig. 2A). *Bromus diandrus* emerged over the span of 11 days, beginning 4 days after planting (Fig. 2B). We observed slightly, but significantly later mean seedling emergence in the herbivory treatment in both *S. pulchra* (exclusion = 6.70 days, herbivory = 6.87 days, *χ* ^2^_(1)_ = 6.12, *P* = 0.013; Fig. 2A) and *B. diandrus* (exclusion = 6.10 days, herbivory = 6.42 days, *χ* ^2^_(1)_ = 9.18, *P* = 0.002; Fig. 2B). Heavier seeds emerged significantly earlier in *S. pulchra* (*χ* ^2^_(1)_ = 45.5, *P* < 0.001; Fig. S3A), but not in *B. diandrus* (*χ* ^2^_(1)_ = 2.23, *P* = 0.14; Fig. S3B). However, there was no interaction between seed mass and caging treatment in *S. pulchra* (*χ* ^2^_(1)_ = 0.87, *P* = 0.35) or *B. diandrus* (*χ* ^2^_(1)_ = 0.30, *P* = 0.59).

### HERBIVORE DAMAGE AND EFFECTS ON MEAN FITNESS

In *S. pulchra*, 151 out of 245 individuals that emerged in the herbivory treatment (61.6%) experienced early‐season damage, and we observed the first herbivore damage in the herbivory treatment after all seedlings had emerged (Fig. 2C). In *B. diandrus*, 206 out of 237 individuals that emerged in the herbivory treatment (86.9%) experienced early‐season damage, and we observed the first herbivore damage in the herbivory treatment on the final day in which seedlings emerged (Fig. 2D). As expected, we observed no evidence of vertebrate herbivore damage in the exclusion treatment. We note that mammalian scat, indicative of herbivore visitation, did not appear in plots until the first day with observed herbivore damage.

In *S. pulchra*, the probability of experiencing early‐season damage in the herbivory treatment was not significantly affected by emergence time or seed mass (Table 1). In *B. diandrus*, individuals in the herbivory treatment that emerged earlier were significantly more likely to experience early‐season damage (Table 1). However, seed mass did not influence the probability of experiencing early‐season damage in this species (Table 1).

**Table 1:** Influence of emergence time and seed mass on the probability of experiencing early‐season damage in the herbivory treatment in *S. pulchra* and *B. diandrus*

	Coefficient ± SE	*df*	χ^2^	*P*
*S. pulchra*				
Emergence time	–0.37 ± 0.24	1	2.44	0.12
Seed mass	–0.18 ± 0.21	1	0.81	0.37
*B. diandrus*				
Emergence time	**–2.38 ± 1.06**	**1**	**5.00**	**0.025**
Seed mass	–0.11 ± 0.28	1	0.17	0.68

Coefficients ± SE are from generalized linear mixed models (GLMMs) of damage (binomial distribution and a logit link) predicted by emergence time and seed mass, with block and maternal line specified as random effects. We evaluated the significance of fixed effects using Type II Wald chi‐square tests. Significant effects (*P* < 0.05) are highlighted in bold.

In *S. pulchra*, herbivory did not significantly affect survival (exclusion = 97.2%, herbivory = 98.4%, *χ* ^2^_(1)_ = 0.74, *P* = 0.39), but significantly reduced mean biomass in surviving individuals (exclusion = 60.4 mg, herbivory = 44.7 mg, *χ* ^2^_(1)_ = 8.74, *P* = 0.003). In *B. diandrus*, herbivory significantly reduced survival (exclusion = 84.5%, herbivory = 58.2%, *χ* ^2^_(1)_ = 43.4, *P* < 0.001), and also significantly reduced mean fecundity among those that survived to produce seeds (exclusion = 362.0 mg, herbivory = 49.5 mg, *χ* ^2^_(1)_ = 282.5, *P* < 0.001).

### HERBIVORY EFFECTS ON PHENOTYPIC SELECTION

**Figure 3:**
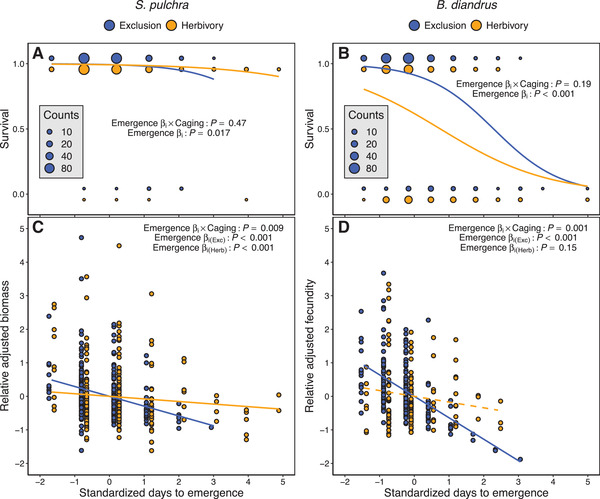
Phenotypic selection on emergence time in caging treatments. We standardized traits across treatments to a mean of 0 and standard deviation of 1 for selection analyses. Solid and dashed lines indicate significant and nonsignificant selection, respectively, and nonsignificant quadratic selection is not depicted (see Table S1). (A and B) Selection on emergence time via survival in (A) *S. pulchra* (*n* = 495) and (B) *B. diandrus* (*n* = 475). Lines represent predicted survival probability based on selection gradients extracted from zero parts of hurdle generalized linear mixed models (GLMMs) (see Table 2), and thus represent direct selection on emergence time. The size of points represents the counts of individuals with a given value of emergence time and survival. Points from each caging treatment with the same values of survival are separated for visibility. (C and D) Selection on emergence time via biomass in (C) *S. pulchra* (*n* = 484) and via fecundity in (D) *B. diandrus* (*n* = 339). Lines represent selection gradients extracted from nonzero hurdle GLMM parts (see Table 2). Points represent relative adjusted biomass/fecundity, which we obtained by adding residuals from nonzero hurdle GLMM parts including linear and quadratic trait terms (divided by mean fitness in each caging treatment) to predicted fecundity/biomass from the selection gradients depicted. Points from each caging treatment with the same values of emergence time are separated for visibility. (D) One *B. diandrus* outlier in the herbivory treatment is not shown (standardized days to emergence = −0.81, relative adjusted fecundity = 13.2).

In both *S. pulchra* and *B. diandrus*, directional selection favored earlier emergence via survival and this selection did not differ significantly between caging treatments (Table 2; Figs. 3A and 3B). However, consistent with our hypothesis, directional selection for earlier emergence via biomass (in *S. pulchra*) and fecundity (in *B. diandrus*) was significantly weaker in the presence of herbivores (Table 2; Figs. 3C and 3D). In *S. pulchra*, there was significant directional selection for earlier emergence via biomass in both caging treatments, whereas in *B. diandrus* significant directional selection for earlier emergence via fecundity was present in the exclusion treatment only (Table 2; Figs. 3C and 3D).

**Table 2:** Estimates of linear (*β*_i_) and quadratic (*γ*_ii_) selection on emergence time and seed mass in the exclusion (Exc) and herbivory (Herb) caging treatments

	Survival						Biomass					
	Linear (*β*_i_)			Quadratic (*γ*_ii_)			Linear (*β*_i_)			Quadratic (*γ*_ii_)		
*S. pulchra*	*β*_i(Exc)_	*β*_i(Herb)_	Δ*β*_i(Herb)_	*γ*_ii(Exc)_	*γ*_ii(Herb)_	Δ*γ*_ii(Herb)_	*β*_i(Exc)_	*β*_i(Herb)_	Δ*β*_i(Herb)_	*γ*_ii(Exc)_	*γ*_ii(Herb)_	Δ*γ*_ii(Herb)_
Emergence time	**–0.98 ± 0.51**	**–0.53 ± 0.62**	0.45	0.11 ± 0.43	–0.052 ± 0.47	–0.16	–0.29* ± 0.069	–0.077* ± 0.083	**0.22**	–0.053 ± 0.057	–0.038 ± 0.061	0.015
Seed mass	1.08 ± 0.58	–0.35 ± 0.78	–1.43	0.91 ± 1.02	0.36 ± 1.16	–0.55	**0.22 ± 0.061**	**0.35 ± 0.073**	0.13	**–0.091 ± 0.039**	**–0.056 ± 0.051**	0.035

	Survival						Fecundity					
	Linear (*β*_i_)			Quadratic (*γ*_ii_)			Linear (*β*_i_)			Quadratic (*γ*_ii_)		
*B. diandrus*	*β*_i(Exc)_	*β*_i(Herb)_	Δ*β*_i(Herb)_	*γ*_ii(Exc)_	*γ*_ii(Herb)_	Δ*γ*_ii(Herb)_	*β*_i(Exc)_	*β*_i(Herb)_	Δ*β*_i(Herb)_	*γ*_ii(Exc)_	*γ*_ii(Herb)_	Δ*γ*_ii(Herb)_
Emergence time	**–1.03 ± 0.26**	**–0.64 ± 0.30**	0.39	0.048 ± 0.16	0.043 ± 0.18	–0.005	–0.64* ± 0.10	–0.17* ± 0.14	**0.47**	–0.096 ± 0.057	0.024 ± 0.11	0.12
Seed mass	0.59 ± 0.27	0.14 ± 0.31	–0.46	0.051 ± 0.21	0.036 ± 0.24	0.015	**0.20 ± 0.067**	**0.091 ± 0.11**	–0.11	–0.013 ± 0.055	–0.055 ± 0.078	–0.042

Values represent coefficients ± SE from hurdle generalized linear mixed models (GLMMs) that evaluated the effects of caging treatment, traits (standardized across treatments to a mean of 0 and standard deviation of 1), and trait × caging treatment interactions on: (1) survival (zero part: binomial GLMM with a logit link); (2) biomass/fecundity of surviving individuals (nonzero part: zero‐truncated negative binomial GLMM with a log link). We included block and maternal line as random effects in both hurdle GLMM parts. Asterisks indicate that selection gradients differed significantly between caging treatments (i.e., significant herbivore‐mediated selection), in which case we tested the significance of selection in each caging treatment separately. Herbivore‐mediated selection is quantified as Δ*β*_i(Herb)_ = *β*_i(Herb)_ – *β*_i(Exc)_ and Δ*γ*_ii(Herb)_ = *γ*_ii(Herb)_ – *γ*_ii(Exc)_. Instances of significant selection (*P* < 0.05) are highlighted in bold. See Table S1 for the full results of Type II Wald chi‐square tests of hurdle GLMM fixed effects.

There was no significant selection on seed mass via survival in either focal species (Table 2; Figs. S4A and S4B). In *S. pulchra*, directional selection favored heavier seeds via biomass with weak, but significant, curvilinearity (Table 2; Fig. S4C). In *B. diandrus*, directional selection similarly favored heavier seeds via fecundity consistently across caging treatments, although quadratic selection was not significant (Table 2; Fig. S4D). All selection gradients and their statistical significance are provided in Table 2. The full results of Type II Wald chi‐square tests of hurdle GLMM fixed effects are provided in Table S1.

## Discussion

In this study, vertebrate herbivory weakened directional selection favoring earlier emergence in two grass species that represent major functional groups in California that differ with respect to origin and life history strategy. This result is consistent with a meta‐analysis that found reduced fitness advantages associated with earlier emergence under field conditions (which presumably included herbivory) versus greenhouse or growth‐chamber conditions lacking natural herbivore communities (Verdú and Traveset 2005). Our study site is dominated by widespread generalist herbivore species; thus, we would expect to see similar patterns in other seasonal herbaceous communities in which generalist vertebrate herbivores impact fitness. Although herbivores have repeatedly been shown to influence selection on flowering phenology (e.g., Pilson 2000; Valdés and Ehrlén 2017), to our knowledge this is the first study to show that herbivores directly alter selection on emergence phenology.

In both focal species, earlier emergence was favored across caging treatments via both survival and subsequent fitness components (biomass in *S. pulchra*; fecundity in *B. diandrus*), with herbivores weakening this selection via the latter fitness components only. The low mortality in *S. pulchra* (compared to *B. diandrus*) was likely a consequence of (1) using biomass rather than fecundity as the subsequent fitness component (it is unlikely that all surviving plants would survive to reproduce) and (2) less intense intraspecific competition due to a more resource‐conservative growth strategy (Dyer and Rice 1999). We expect that competition was the main factor driving selection for earlier emergence in both species. In *Brassica rapa* grown at lower densities than those used in this study, the contribution of competition to selection favoring earlier emergence was five times greater than that of the general abiotic environment (Weis et al. 2015). In our study, herbivory did not increase relative survival probability for later‐emerging individuals but did increase the relative fecundity/biomass of later‐emerging individuals that survived. One potential reason for this difference is that any survival benefits of weakened competition due to herbivory in later‐emerging individuals were outweighed by mortality due to other factors, such as drought (Potts et al. 2012) or pathogens (Malmstrom et al. 2005).

We proposed several mechanisms by which vertebrate herbivory could weaken selection for earlier emergence, and our results suggest that the importance of each mechanism differed between the two focal species. Escaping herbivory by remaining in the soil did not contribute substantially, if at all, to observed results in either species because the first instances of herbivore damage occurred after almost every individual had emerged. This mechanism is likely to be more important for populations with greater variance in emergence time, resulting in comparatively longer exposure to herbivores in the earliest‐emerging individuals. In *B. diandrus*, earlier‐emerging plants were more likely to experience early‐season damage, consistent with greater apparency leading to selective grazing (cf. Louthan et al. 2014; Thomann et al. 2018). In *S. pulchra*, emergence time did not affect the probability of experiencing early‐season damage, suggesting that selective grazing of more apparent earlier‐emerging plants was not an important mechanism driving weaker selection for earlier emergence in this species. Instead, the main effect of vertebrate grazing in *S. pulchra* may have been to lower the light limitation of smaller plants (cf. Borer et al. 2014), thus reducing the competitive disadvantage associated with later emergence. However, we could only evaluate the presence/absence of damage for the earlier part of the growing season, thus we have only a snapshot view of the mechanisms by which herbivores influenced selection. Although early‐season herbivory can be most impactful (Waterton and Cleland 2016), unmeasured later‐season herbivory may also have influenced patterns of selection. For example, mammalian grazing favors less apparent, shorter‐scaped inflorescence morphs of the perennial herb *Primula farinosa* (Thomann et al. 2018), and similar late‐season herbivory on *B. diandrus* inflorescences might have favored smaller, less apparent individuals. Further research should characterize the importance of these mechanisms and determine how they are affected by seasonal variation in vertebrate herbivore diet and activity (e.g., Batzli and Pitelka 1971).

As with any experiment, the patterns of selection that we observed in this study may have been influenced by the experimental methods. First, because we used field‐collected seeds, variation in maternal environments within source populations likely influenced seed mass, emergence time, fitness, and the relationships between them (Roach and Wulff 1987). Although seed mass is a measure of maternal provisioning, which is itself influenced by the maternal environment (Halpern 2005), we did not account for other nonprovisioning effects of variation in maternal environment such as epigenetic inheritance (Henderson and Jacobsen 2007). Second, although cages in each treatment intercepted similar amounts of PAR, cage openings may have delayed emergence in the herbivory treatment by affecting soil moisture or temperature (Baskin and Baskin 2014). The greater proportion of late‐emerging plants in the herbivory treatment should result in greater competitive advantages for the earliest‐emerging individuals compared to the exclusion treatment. Thus, if anything, we expect such delays in emergence would counteract the weakening of directional selection resulting from vertebrate herbivory. Third, we watered plots to avoid mass mortality due to drought; this may have intensified selection for earlier emergence because priority effects are typically stronger in more benign resource environments (Vannette and Fukami 2014). Finally, for the perennial *S. pulchra*, estimates of selection based on biomass after one growing season may not reflect selection when evaluated over the lifetime of each plant, particularly if there is a nonlinear relationship between first‐year biomass and future survival and fecundity. These relationships might further depend on how herbivore damage influences growth and investment in reproduction in subsequent years (Puentes and Ågren 2012). Regardless, the first growing season is a critical window for establishment of perennial species with disproportionate influence on population persistence (Grubb 1977).

Our results have implications for the adaptive potential of seasonal herbaceous species under current and future global change. As with other phenological traits, such as flowering phenology (Anderson et al. 2012), emergence timing is expected to evolve with climate change (Walck et al. 2011; Cochrane et al. 2015). Genetic variation will be a key factor determining the potential for *in situ* adaptive evolution (Walck et al. 2011; Cochrane et al. 2015). Despite negatively impacting plant fitness directly, our results suggest that vertebrate herbivory could reduce the rate at which competition can deplete genetic variation in emergence time. Additionally, many plant species are rapidly evolving to advance their seasonal phenologies with climate change. For example, earlier flowering has evolved in concert with rising temperatures (Anderson et al. 2012), and populations have shifted toward earlier emergence in response to drought (Dickman et al. 2019). In such cases, vertebrate herbivory could impede adaptive responses toward earlier phenologies, which in turn could have negative impacts on population persistence (Cleland 2012). Alternatively, herbivory might act to protect populations from maladaptive evolutionary responses, such as evolving earlier spring phenology that exposes plants to greater risk of frost damage (Inouye 2008; Iler et al. 2019). Consequently, in weakening selection for earlier emergence, herbivores may have variable effects on population persistence in the face of global environmental change.

## AUTHOR CONTRIBUTIONS

JW and EEC conceived the ideas and designed the methodology. JW collected and analyzed the data, and led the writing of the manuscript.

## DATA ARCHIVING

Data, metadata, and R code are available on the Open Science Framework: https://doi.org/10.17605/OSF.IO/4JNCT.

Associate Editor: A. Charmantier

## Supporting information




**Table S1**. Type II Wald chi‐square tests of hurdle generalized linear mixed models (GLMMs) for significant linear and quadratic phenotypic selection in caging treatments.
**Figure S1**. Monthly precipitation at the experiment site for 2017–2018 and the 30‐year average from July 1988 ‐ June 2018.
**Figure S2**. Influence of seed mass on emergence probability in caging treatments.
**Figure S3**. Influence of seed mass on emergence time in caging treatments.
**Figure S4**. Phenotypic selection on seed mass in caging treatments.
