# Supplementary material for: Vertebrate herbivory weakens directional selection for earlier emergence in competition
Source: Evol Lett. 2021 Mar 25;5(3):265–76. doi: 10.1002/evl3.222 (PMC8190447; doi:10.1002/evl3.222)
Supplement: Supplementary file 1 — Table S1. Type II Wald chi‐square tests of hurdle generalized linear mixed models (GLMMs) for significant linear and quadratic phenotypic selection in caging treatments. Figure S1. Monthly precipitation at the experiment site for 2017–2018 and the 30‐year average from July 1988 ‐ June 2018. Figure S2. Influence of seed mass on emergence probability in caging treatments. Figure S3. Influence of seed mass on emergence time in caging treatments. Figure S4. Phenotypic selection on seed mass in caging treatments. [file EVL3-5-265-s001.docx]

**Supporting Information**

**Vertebrate herbivory weakens directional selection for earlier emergence in competition**

Joseph Waterton and Elsa E. Cleland

**Supplemental Methods**

*Herbivore exclusion experiment*

We collected *S. pulchra* seeds in Sedgwick Reserve from a naturally occurring population on the Byrne observatory mesa (34.692° N 120.043° W). We collected *B. diandrus* seeds in Elliott Chaparral Reserve from a naturally occurring population several hundred yards west of the Nutrient Network site (32.889° N, 117.091° W).

We planted seeds with radicles oriented downwards at a depth of 1 cm and next to toothpicks to aid identification of non-emerged individuals (these were gently removed after the corresponding individual had emerged). Immediately after planting all seeds, we applied 1.5 l of water evenly across plots (equivalent to approximately 4 mm precipitation) to initiate germination. We applied this same volume of water daily until 21 February, on which we observed the first rain event after planting. Following this, we applied 2.5 l of water to all plots (equivalent to approximately 6 mm precipitation) each Wednesday and Friday in weeks without forecasted rain events to minimize the risk of mass mortality due to drought. We continually removed large weeds around subplots so that focal plants could be monitored. We stopped all supplemental watering after 9 May, by which point all *B. diandrus* that had flowered had begun to senesce. Due to the density of individuals in subplots, we were unable to record *B. diandrus* flowering dates.

**Table S1.** Type II Wald chi-square tests of hurdle generalized linear mixed models (GLMMs) for significant linear (β_i_) and quadratic (γ_ii_) phenotypic selection in the exclusion (Exc) and herbivory (Herb) caging treatments. We included block and maternal line as random effects in both hurdle GLMM parts. Significant trait × caging treatment interactions indicate that selection differs between treatments, in which case we tested the significance of selection in separate models for each treatment. Significant effects (*P* < 0.05) are highlighted in bold.

| **Trait term** | ***S. pulchra*** | | | | | | | |
| --- | --- | --- | --- | --- | --- | --- | --- | --- |
|  | **Survival** | | | | **Biomass** | | | |
|  | **Main** | **Interaction** | **Exc** | **Herb** | **Main** | **Interaction** | **Exc** | **Herb** |
| Emergence time (β_i_) | **χ^2^_(1)_ = 5.72**  ***P* = 0.017** | χ^2^_(1)_ = 0.52  *P* = 0.47 | - | - | **χ^2^_(1)_ = 12.2**  ***P* < 0.001** | **χ^2^_(1)_ = 6.75**  ***P* = 0.009** | **χ^2^_(1)_ = 16.6**  ***P* < 0.001** | **χ^2^_(1)_ = 18.1**  ***P* < 0.001** |
| Seed mass (β_i_) | χ^2^_(1)_ = 0.62  *P* = 0.43 | χ^2^_(1)_ = 3.40  *P* = 0.065 | - | - | **χ^2^_(1)_ = 31.5**  ***P* < 0.001** | χ^2^_(1)_ = 3.20  *P* = 0.074 | - | - |
| Emergence time (γ_ii_) | χ^2^_(1)_ = 0.015  *P* = 0.90 | χ^2^_(1)_ = 0.11  *P* = 0.74 | - | - | χ^2^_(1)_ = 3.75  *P* = 0.053 | χ^2^_(1)_ = 0.057  *P* = 0.81 | - | - |
| Seed mass (γ_ii_) | χ^2^_(1)_ = 1.00  *P* = 0.32 | χ^2^_(1)_ = 0.22  *P* = 0.64 | - | - | **χ^2^_(1)_ = 6.21**  ***P* = 0.013** | χ^2^_(1)_ = 0.46  *P* = 0.50 | - | - |
| **Trait term** | ***B. diandrus*** | | | | | | | |
|  | **Survival** | | | | **Fecundity** | | | |
|  | **Main** | **Interaction** | **Exc** | **Herb** | **Main** | **Interaction** | **Exc** | **Herb** |
| Emergence time (β_i_) | **χ^2^_(1)_ = 19.5**  ***P* < 0.001** | χ^2^_(1)_ = 1.68  *P* = 0.19 | **-** | **-** | **χ^2^_(1)_ = 30.5**  ***P* < 0.001** | **χ^2^_(1)_ = 10.8**  ***P* = 0.001** | **χ^2^_(1)_ = 37.6**  ***P* < 0.001** | χ^2^_(1)_ = 2.09  *P* = 0.15 |
| Seed mass (β_i_) | χ^2^_(1)_ = 2.67  *P* = 0.10 | χ^2^_(1)_ = 2.17  *P* = 0.14 | - | - | **χ^2^_(1)_ = 8.90**  ***P* = 0.003** | χ^2^_(1)_ = 0.95  *P* = 0.33 | - | - |
| Emergence time (γ_ii_) | χ^2^_(1)_ = 0.26  *P* = 0.61 | χ^2^_(1)_ = 0.001  *P* = 0.98 | - | - | χ^2^_(1)_ = 1.77  *P* = 0.18 | χ^2^_(1)_ = 1.15  *P* = 0.28 | - | - |
| Seed mass (γ_ii_) | χ^2^_(1)_ = 0.12  *P* = 0.73 | χ^2^_(1)_ = 0.004  *P* = 0.95 | - | - | χ^2^_(1)_ = 0.75  *P* = 0.39 | χ^2^_(1)_ = 0.29  *P* = 0.59 | - | - |

**
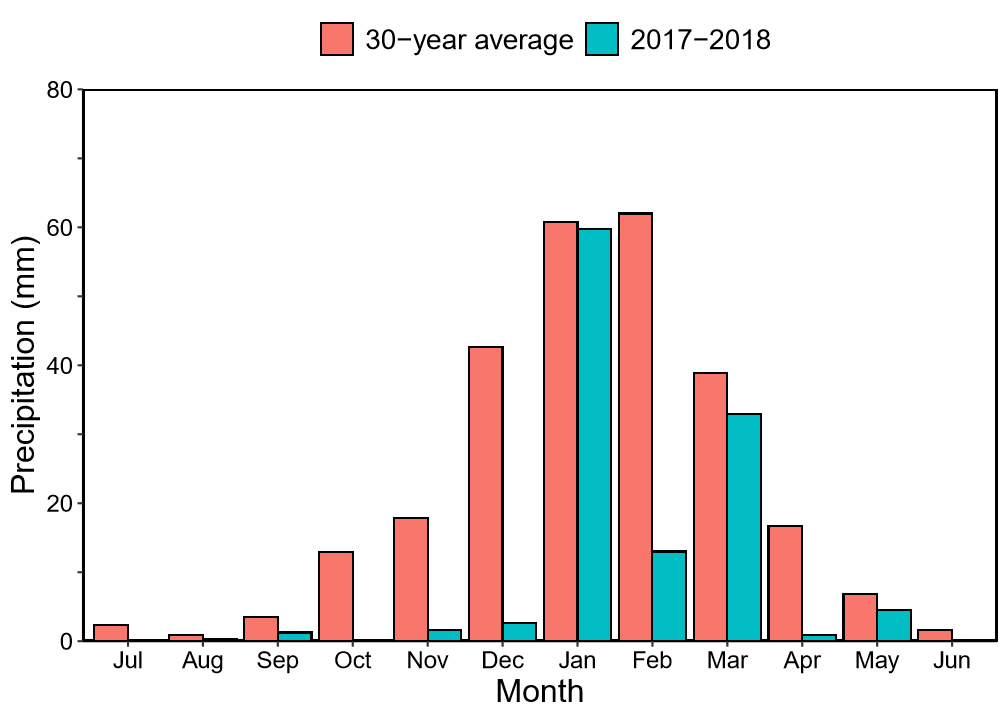
**

**Figure S1.** Monthly precipitation at the experiment site for 2017-2018 and the 30-year average from July 1988 - June 2018. We retrieved precipitation data from the PRISM Climate Group database (prism.oregonstate.edu/).


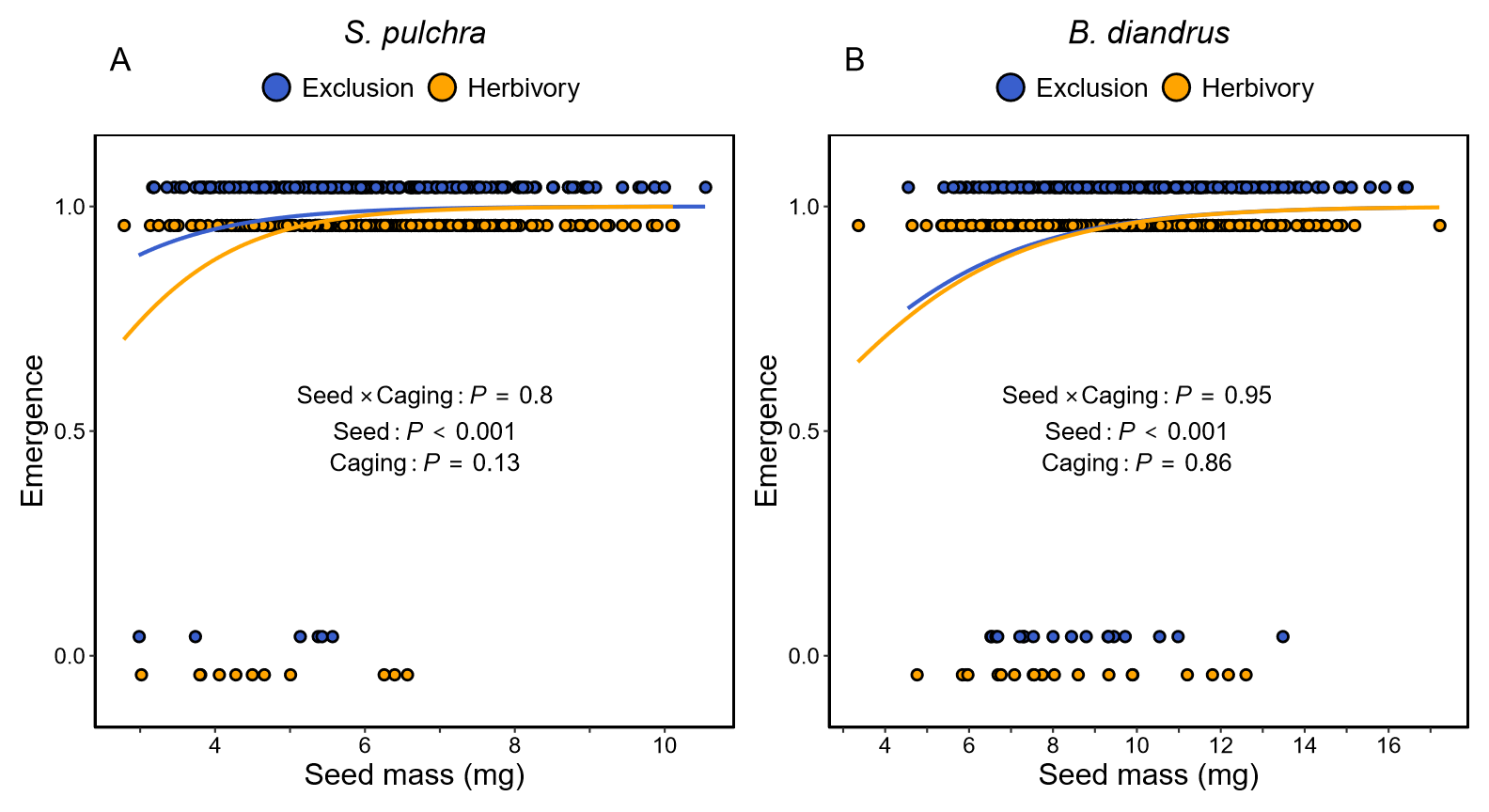


**Figure S2.** Influence of seed mass on emergence probability in caging treatments in (A) *S. pulchra* (*n* = 512) and (B) *B. diandrus* (*n* = 512). *P*-values are from generalized linear mixed models of emergence (binomial distribution and a logit link) predicted by caging treatment, seed mass, and their interaction, with block and maternal line specified as random effects. Points from each caging treatment with the same values of emergence are separated for visibility. Solid lines indicate a significant regression and dashed lines indicate a non-significant regression.

**
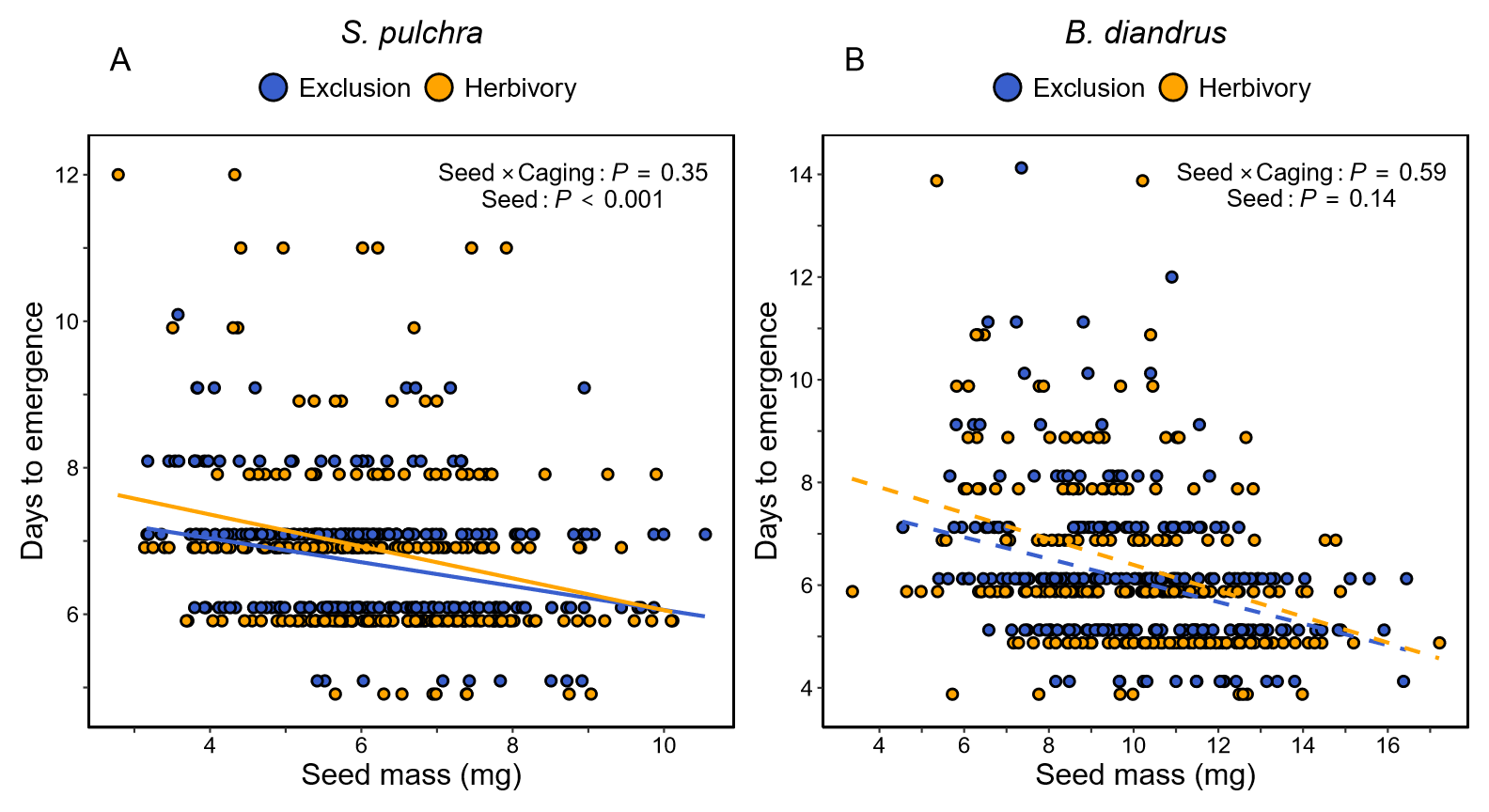
**

**Figure S3.** Influence of seed mass on emergence time in caging treatments in (A) *S. pulchra* (*n* = 495) and (B) *B. diandrus* (*n* = 475). *P*-values are from linear mixed models of emergence time (log-transformed) predicted by caging treatment, seed mass, and their interaction, with block and maternal line specified as random effects. Points from each caging treatment with the same days to emergence have been separated for visibility. Solid lines indicate a significant regression and dashed lines indicate a non-significant regression.

**
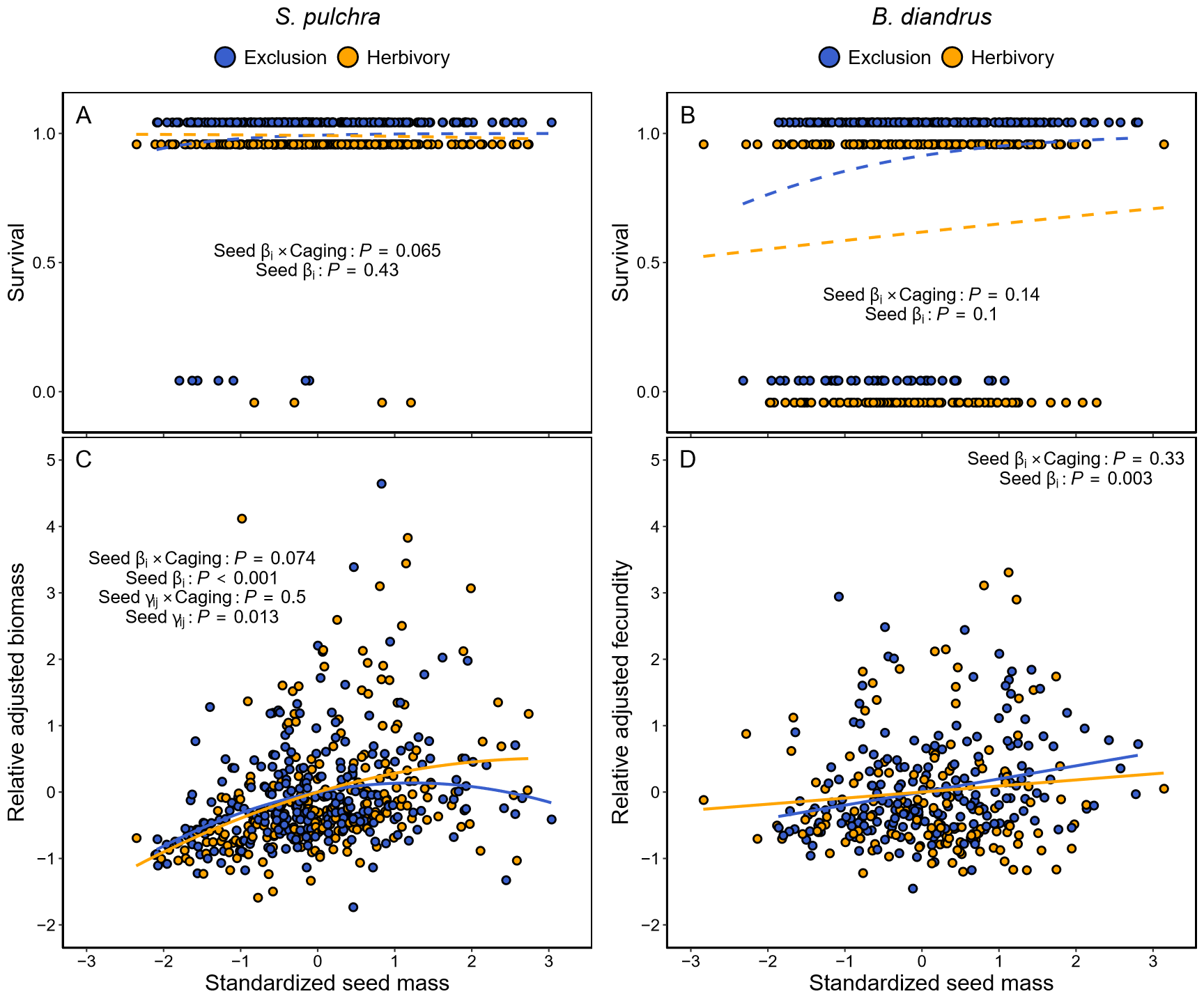
**

**Figure S4.** Phenotypic selection on seed mass in caging treatments. We standardized traits across treatments to a mean of 0 and standard deviation of 1 for selection analyses. Solid and dashed lines indicate significant and non-significant selection, respectively, and non-significant quadratic selection is not depicted (see Table S1). (A, B) Selection on seed mass via survival in (A) *S. pulchra* (*n* = 495) and (B) *B. diandrus* (*n* = 475). Lines represent predicted survival probability based on selection gradients extracted from zero parts of hurdle generalized linear mixed models (GLMMs) (see Table 2), and thus represent direct selection on seed mass. Points from each caging treatment with the same values of survival are separated for visibility. (C, D) Selection on seed mass via biomass in (C) *S. pulchra* (*n* = 484) and via fecundity in (D) *B. diandrus* (*n* = 339). Lines represent selection gradients extracted from non-zero hurdle GLMM parts (see Table 2). Points represent relative adjusted biomass/fecundity, which we obtained by adding residuals from non-zero hurdle GLMM parts including linear and quadratic trait terms (divided by mean fitness in each caging treatment) to predicted fecundity/biomass from the selection gradients depicted. (D) One *B. diandrus* outlier in the herbivory treatment is not shown (standardized seed mass = 0.81, relative adjusted fecundity = 13.1).
